# The prognostic significance of immune microenvironment in breast ductal carcinoma in situ

**DOI:** 10.1038/s41416-020-0797-7

**Published:** 2020-03-17

**Authors:** Michael S. Toss, Asima Abidi, Dorothea Lesche, Chitra Joseph, Sakshi Mahale, Hugo Saunders, Tanjina Kader, Islam M. Miligy, Andrew R. Green, Kylie L. Gorringe, Emad A. Rakha

**Affiliations:** 1 Nottingham Breast Cancer Research Centre, Division of Cancer and Stem Cells, School of Medicine, The University of Nottingham, Nottingham City Hospital, Nottingham, UK; 2 Histopathology Department, South Egypt Cancer Institute, Assiut University, Assiut, Egypt; 3 Cancer Genomics Program, Peter MacCallum Cancer Centre, Melbourne, Australia; 4 The Sir Peter MacCallum Department of Oncology, University of Melbourne, Parkville, Australia

**Keywords:** Breast cancer, Cancer

## Abstract

**Background:**

The role of different subtypes of tumour infiltrating lymphocytes (TILs) in breast ductal carcinoma in situ (DCIS) is still poorly defined. This study aimed to assess the prognostic significance of B and T lymphocytes and immune checkpoint proteins expression in DCIS.

**Methods:**

A well characterised DCIS cohort (*n* = 700) with long-term follow-up comprising pure DCIS (*n* = 508) and DCIS mixed with invasive carcinoma (IBC; *n* = 192) were stained immunohistochemically for CD20, CD3, CD4, CD8, FOXP3, PD1 and PDL1. Copy number variation and TP53 mutation status were assessed in a subset of cases (*n* = 58).

**Results:**

CD3+ lymphocytes were the predominant cell subtype in the pure DCIS cohort, while FOXP3 showed the lowest levels. PDL1 expression was mainly seen in the stromal TILs. Higher abundance of TILs subtypes was associated with higher tumour grade, hormone receptor negativity and HER2 positivity. Mutant TP53 variants were associated with higher levels of stromal CD3+, CD4+ and FOXP3+ cells. DCIS coexisting with invasive carcinoma harboured denser stromal infiltrates of all immune cells and checkpoint proteins apart from CD4+ cells. Stromal PD1 was the most differentially expressed protein between DCIS and invasive carcinoma (*Z* = 5.8, *p* < 0.0001). Dense TILs, stromal FOXP3 and PDL1 were poor prognostic factors for DCIS recurrence, while dense TILs were independently associated with poor outcome for all recurrences (HR = 7.0; *p* = 0.024), and invasive recurrence (HR = 2.1; *p* = 0.029).

**Conclusions:**

Immunosuppressive proteins are potential markers for high risk DCIS and disease progression. Different stromal and intratumoural lymphocyte composition between pure DCIS, DCIS associated with IBC and invasive carcinoma play a potential role in their prognostic significance and related to the underlying genomic instability. Assessment of overall TILs provides a promising tool for evaluation of the DCIS immune microenvironment.

## Background

The incidence of breast ductal carcinoma in situ (DCIS) increased dramatically after the introduction of mammographic screening and accounts up to 25% of the screen detected breast cancers in the UK with similar rates in the US.^1,2^ However, management of DCIS remains as a clinical challenge with a considerable proportion of patients are either over- or under-treated due to the lack of a robust prognostic assessment model to stratify patients’ risk. Current management decisions rely on conventional clinicopathological parameters as the clinical utility of available gene expression assays and molecular prognostic biomarkers remain to be demonstrated. It is hence important to identify other robust markers for DCIS for behaviour predilection to decide a personalised treatment approach.

Although the role of tumour microenvironment in disease behaviour is undeniable, incorporation of the microenvironmental factors with the clinicopathological and/or molecular signature for DCIS risk assessment is still limited. The tumour immune microenvironment undergoes several changes during carcinogenesis.^3^ The triggering of an immune response in the form of infiltration of lymphocytes and other inflammatory mediators is a phenomenon known as “cancer immuno-editing”.^4^ It is known that subtype, density and location of tumour infiltrating lymphocytes (TILs) play a role in cancer prognosis. Infiltration of T lymphocytes extends an anti-tumour response through the CD8+ cytotoxic T lymphocytes while an immunosuppressive response occurs through CD4+ FOXP3+ regulatory T cells (Tregs).^5,6^ Similarly, CD20+ B lymphocytes are associated with a humoral anti-tumour response.^7^ The role of TILs in invasive breast cancer (IBC) is well validated, with a high TILs density associated with better response to adjuvant or neoadjuvant therapy and favourable outcome especially in triple negative (TNBC) and HER2+ subtypes.^8–13^ However, there are conflicting data for the role of TILs in DCIS prognosis with some studies claiming an unfavourable role^14^ while others did not find association with the outcome.^15,16^

An important component of immuno-editing is the development of an immunosuppressive microenvironment mediated through the activation of the immune checkpoint pathway. The programmed cell death 1 (PD1) protein, a member of the B7/CD28 family of co-stimulatory receptors present mainly on T cells, sends inhibitory signals to T cells to suppress the anti-tumour response.^4,17^ PD1 functions mainly through its ligand PDL1, which is expressed on a variety of immune cells such as antigen presenting cells and activated T and B lymphocytes and tumour cells.^18,19^ Revolutionary results have been achieved from clinical trials involving monoclonal antibodies that target PD1 and block the PD1/PDL1 pathway in patients with non-small cell lung cancer, melanoma and renal cancer.^20^

In a previous study we have demonstrated the prognostic value of TILs, as assessed on H&E stained slides, in DCIS.^14^ Here we hypothesised that various immune cell subpopulations and the immune checkpoint proteins play roles in DCIS behaviour and this can be related to the underlying molecular prolife. In this study, we evaluated the expression and the prognostic significance of T and B lymphocytes and immune checkpoint proteins PD1 and PDL1 in a large well characterised DCIS cohort with long-term follow-up. We also investigated the relationship between genomic copy number alteration and *TP53* variants (as measures of genomic instability) with immune cell infiltration in a subset of cases.

## Methods

### Study Cohort

This study was conducted on a well characterised annotated large DCIS cohort, comprising primary pure DCIS cases and DCIS associated with invasive tumour (DCIS-mixed) diagnosed at Nottingham City Hospital, Nottingham, UK, between 1990 to 2012 as described.^21^ The clinicopathological characteristics of the study cohort are summarised in Supplementary Table 1. The patients’ demographic data, histopathological parameters, overall TILs density, DCIS management including post-operative radiotherapy (RT) and development of local recurrence along with patient outcome was available. Tumour grade was classified according to the three-tier system; low, intermediate and high grades.^22^ Local recurrence free interval (LRFI) was defined as the time interval (in months) between 6 months after the primary DCIS surgery and occurrence of ipsilateral local recurrence either as DCIS or IBC. Recurrence was considered when it occurred in the same quadrant and showed a similar nuclear grade or higher than the primary tumour. In this study, cases with new lesions in other quadrants, or with lower nuclear grade and tumours developing in the contralateral breast were not counted as a recurrence and were censored at time of development of the new event. All recurrence events were checked three times by three researchers with pathology experience. Moreover, they were histologically reviewed to ensure that the morphology matches that stated in the original histology. We did our best to differentiate true recurrences from new primary tumours with acknowledgement of the limitations of our ability to accurately make such a distinction. We are interested in the behaviour of DCIS under investigation rather than the risk of other events that happened following the primary diagnosis of DCIS, either new primary ipsilateral or contralateral events. We have not included these events in our analysis to avoid the confounding effects of these tumours, which may have different biological characteristics features.

Data on Oestrogen Receptor (ER), Progesterone Receptor (PR), Human Epidermal Growth Factor Receptor 2 (HER2), Ki-67, TP53, CK5/6, and HIF-1α were available to this cohort as previously described. Briefly, ER and PR positivity were both defined as ≥1% nuclear tumour staining.^23^ Immunoreactivity of was scored using HercepTest guidelines. Chromogenic in situ Hybridisation (*CISH*) was used to assess *HER2* gene amplification in cases with 2+ score using the HER2 CISH pharmDx™ kit (Dako).^24^ Proliferation index was evaluated through Ki-67 antibody immunohistochemical (IHC) staining and defined as high when ≥14% of cells showed nuclear positivity.^25^ The molecular subtypes were classified as: Luminal A (ER+ and/or PR+, HER2− and Ki67 low), Luminal B (ER+ and/or PR+, HER2− and Ki67 high or ER+ and HER2+), HER2 enriched (HER2+ and ER−) and Triple Negative (ER−, PR− and HER2−). TP53 and CK5/6 proteins were assessed immunohistochemically, and the percentage of positive nuclear and membranous expression were scored, respectively. More than 1% HIF-1α nuclear immunoreactivity was considered positive.^26,27^ High TIL density was considered where the average number lymphocytes/duct was 20 or more.^14^

### Immunohistochemistry of immune cell markers and checkpoint proteins

Tissue microarrays (TMAs) were prepared from both cohorts using a TMA Grand Master 2.4-UG-EN machine (3D Histech) with 1 mm punch sets. The TMA was prepared from all representative areas of heterogeneous morphology and grade, when present. Apart from the TMAs, 30 full-face sections were also assessed to reveal the infiltration pattern of various TILs subtypes and immune checkpoint proteins.

Immunohistochemical staining (IHC) was performed on TMA sections using a Novocastra Novolink TM Polymer Detection Systems Kit (Code: RE7280-K, Leica, Biosystems, UK). Primary antibody specific for CD20 (mouse monoclonal, DAKO Clone L26/M0755), CD3 (rabbit monoclonal antibody [SP7], Abcam (ab16669)), CD8 (mouse monoclonal, DAKO Clone C8/144B), CD4 (rabbit monoclonal antibody [EPR6855], Abcam (ab133616)), FOXP3 (mouse monoclonal [236A/E7), Abcam (ab20034)), PD1 (Mouse monoclonal [EH33] Cell signalling (43248S)), PDL1 (Rabbit monoclonal antibody [E1L3N] Cell signalling (13684S)) were used to characterise TILs and immune checkpoint proteins. Supplementary Table 2 summarises the details of the antibodies used for IHC staining. Sections from normal tonsil were used as a positive control while a negative control was generated by omitting the primary antibodies.

### Immunoscoring

Stained slides were digitally scanned using a Nannozoomer (Hamamatsu Photonics), at ×20 magnification high resolution images and viewed using the Aperio ImageScope Viewer software version 12.3.3 (developed by Leica Biosystems Imaging). All cases were scored visually using the digitised images for stromal expression of TILs as previous described in ref. ^14^, and for intratumour TILs. Briefly, the average number of lymphocytes touching DCIS basement membrane or away from it by one lymphocyte cell thickness was counted. In addition, the average number of intratumour TILs per duct was counted for CD20, CD3, CD4, CD8, FOXP3 and PD1. PDL1 expression in tumour epithelial cells was scored as percent of cells showing membranous and/or cytoplasmic staining, where cases with >1% positive cells were considered positive.^15^ The pure DCIS and the in situ component of mixed cases which clearly defined a demarcated tumour edge were assessed, separately. Average score was used as a final score for cases with multiple cores. All cases were scored independently by two observers (A Abidi and M Toss) blinded to patient histopathological data and clinical outcome. The average score was considered in discrepant cases between the two observers.

### DNA Extraction, targeted sequencing library preparation, enrichment, and sequencing

Sections from formalin-fixed paraffin-embedded (FFPE) blocks from a subset of DCIS cases (*n* = 58) were reviewed by two pathologists to select representative sections. The cases were randomly selected based on tissue block availability and adequacy of tumour tissue for DNA extraction. Tumour epithelial cells were needle-microdissected from 10 µm sections (8 – 20 slides) after haematoxylin staining and DNA was isolated using the AllPrep DNA/RNA FFPE kit (Qiagen, Hilden, Germany) according to the manufacturer’s protocol. Targeted sequencing of tumour DNA was performed using an Agilent SureSelect XT Custom Panel (Agilent, Santa Clara, CA, USA) which targeted breast cancer specific genes including *TP53*. Library preparation was performed from at least 80 ng of tumour DNA using the KAPA Hyper system (Agilent). For samples with lower DNA yields available (≤ 10 ng), library preparation was performed with the NEBNext® Ultra™ II DNA Library Prep Kit (New England BioLabs® Inc, Ipswich, MA, USA) and used for low-coverage whole-genome sequencing (LC-WGS) as described previously.^28^ Illumina Nextseq500 (paired-end 75 bp reads) was used to run pooled, normalised, indexed libraries according to Illumina protocols. Average sequencing depth was 400×(SureSelect) and 1.3×(LC-WGS).

### Copy number and variant data analysis

Data was processed by an in-house bioinformatics pipeline at the Peter MacCallum Cancer Centre (Melbourne) to detect and filter for high confidence somatic variants. Paired‐end sequence reads were aligned to the g1 k v37 hg19 reference genome using BWA. Optical duplicate reads were removed using Picard and the Genome Analysis Tool Kit (GATK) was used to perform local realignment around indels and base quality score recalibration in accordance with the recommended best practice workflow.^29^ Single nucleotide polymorphism (SNP) and indel variants were called using GATK Unified Genotyper, Platypus,^30^ and Varscan 2.^31^ Called variants were annotated using the Ensembl Variant Effect Predictor.^32^ Somatic mutations were identified by removing previously available germline variant data for hereditary breast and ovarian cancer panel genes and by applying the following filters; canonical transcript; bidirectional read; quality ≥ 100; variants identified by at least two variant callers; minor allele frequency (MAF) present at ≤0.0001 in ExAc (Version 0.3.1, excluding TCGA data),^33^ GnomAD (Version 2.0), EVS (Version ESP6500SI‐V2‐SSA137) [Exome Variant Server, available http://evs.gs.washington.edu/EVS/], and 1000 Genomes^34^ databases. Genomics regions identified by Scheinin et al^35^ were removed from analysis as described previously.^28^

Copy number profiles were obtained using the SureSelect panel off-target reads with CopywriteR^36^ and Control-FREEC for LC-WGS reads.^37^ A lymphocyte DNA control run (NA12878, Coriell Institute) in the same sequencing batch was used for baseline normalisation. Data were then imported to NEXUS Copy Number™ (software v9.0; BioDiscovery Inc, El Segundo, CA, USA), segmented using a FASST2 segmentation algorithm and visualised using a circular binary segmentation algorithm to determine gains and losses as described.^38^ Fraction of the genome altered (FGA) was calculated as the percentage of base pairs with copy number gain or loss. Number of telomeric allelic imbalances (NTAI) was calculated as the count of telomeres (excluding chromosome Y) with a gain or loss.^15^ Twenty cases showed deleterious/missense *TP53* variants annotated as described above.

### Statistical analysis

All statistical tests were conducted on IBM SPSS software (version 24.0, Chicago, IL, USA) for Windows. For the dichotomisation of the immune cells and checkpoint protein densities, median values were used as cut off points.

Spearman’s Rho test was used to correlate between the expression of different immune cells with each other, overall TILs and with the proliferation marker (Ki67), TP53 and HIF-1α. Chi square test was used to determine the association between different markers densities with other clinicopathological parameters. Expression of PDL1 within the tumour cells was not included in the analysis as the percentage of positive cases was very low (2% only). Mann–Whitney U test and Kruskal-Wallis test were used to compare between TILs densities and FGA, NTAI and *TP53* variants. Differential expression of various study markers between pure DCIS and DCIS coexisting with IBC was analysed using the continuous score data and the Mann–Whitney *U* test. The association with LRFI was assessed in univariate analysis using Kaplan–Meier curves and Log Rank Test. Cox regression was carried out for multivariate analysis to adjust for confounding by conventional prognostic factors. Survival analyses were confined to patients treated with breast conserving surgery (BCS) with or without radiotherapy as it was the highest group showing ipsilateral recurrence (>95% of recurrences) and the smaller number of events in patients treated with mastectomy. For each test conducted, a two-tailed *p*-value of <0.05 was considered statistically significant.

## Results

### Patterns of expression and distribution of various inflammatory cells and immune checkpoints

The assessment of the full-face sections revealed that TILs infiltration was comparable to the TMA sections thus justifying the representability of the TMA sections for immunoscoring both in the stromal and intratumoural compartments. Uninformative cores (lost, folded tissue or cores containing <15% tumour cells and/or stroma) were excluded from scoring.

**Fig. 1:**
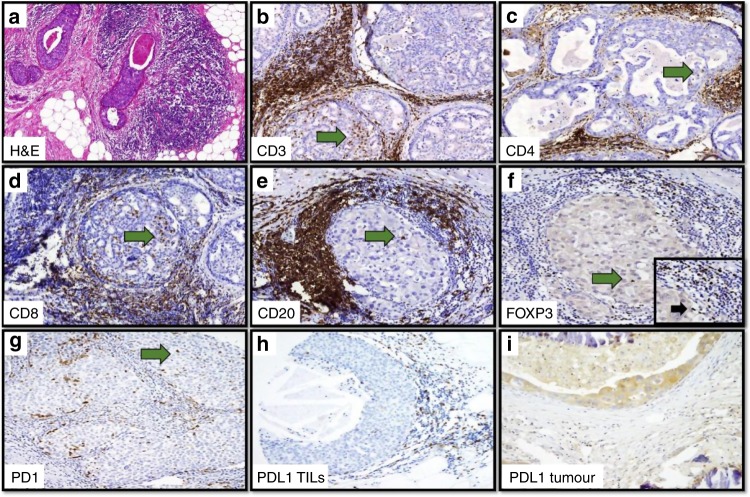
Photomicrograph for immune cells and checkpoint proteins in DCIS. **a** Dense Overall TILs infiltration in H&E stained sections (×20). Dense stromal and intratumoural infiltration of various immune cell markers, **b** CD3, **c** CD4, **d** CD8, **e** CD20, **f** FOXP3, **g** PD1 and **h** PDL1. Expression of PDL1 within the tumour epithelial cells is shown in (I). Green arrows highlight the intratumoural lymphocytes. Inset: higher magnification (×40) showing stromal FOXP3 positive cells.

Supplementary Table 3 summarises the distribution of various types of lymphocytes as well as PD1 and PDL1 expression either in the stroma or intratumourally. The predominant lymphocytes in DCIS were CD3+ cells either intratumourally (median 1 cell/duct, range 0–50) or in the stroma (median 3 cells/duct, range 0–100) whereas CD4+ cells were the most frequent subtype of T cells (range 0–60 in the stroma and 0–25 in the intratumoural compartment). Stromal FOXP3+ lymphocytes were the least frequent (median 0 cells/duct, range 0–25) while PD1+ cells were the lowest type intratumourally (median 0 cells/duct, range 0–15). Only 2% of pure DCIS cases showed positive expression for PDL1 within tumour cells. Fig. 1 and Supplementary Fig. 1 show examples of dense TIL infiltrates in H&E and IHC stained sections with various markers demonstrating the distribution of immune cells and immune checkpoint protein in pure DCIS.

**Fig. 2:**
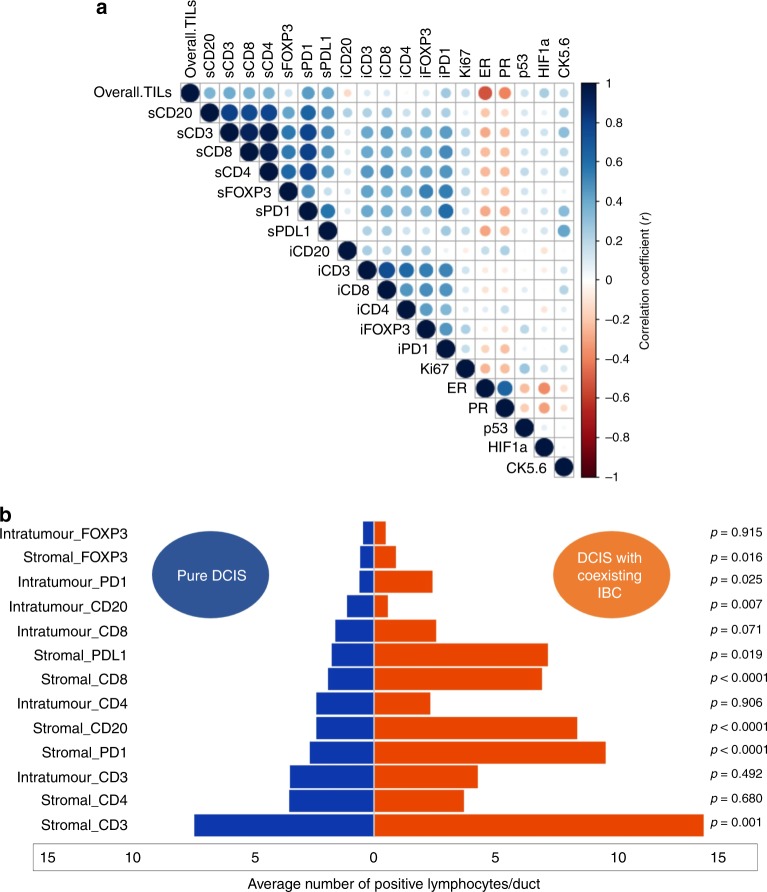
Distribution of various TILs subtypes in pure and mixed DCIS cohorts. **a** Correlation Matrix showing the correlation between TILs subtypes, overall TILs, and other associated biomarkers. (s stromal, i intratumoural). Blue colour refers to positive correlation, while the red colour reflects negative correlations. The intensity of the colour propionate to the correlation coefficient. **b** Tornado (Butterfly) plot showing the differential expression level of various lymphocytes between pure DCIS and DCIS mixed with synchronous invasive breast cancer (IBC).

Stromal CD3+, CD8+, CD4+, PDL1+ and PD1+ lymphocytes showed the highest correlation with overall TILs (all *r* > 0.4, *p* < 0.0001). The strongest correlation between various lymphocytes was observed with CD3+ and CD4+ lymphocytes (*r* = 0.96, *p* < 0.0001), followed by CD4+ and CD8+ cells (*r* = 0.94, *p* < 0.0001). Fig. 2a shows a correlation matrix that summarises the associations between overall TILs, various immune cell subtypes, PD1, PDL1, hormonal receptors, Ki67, HIF-1α, CK5/6 and p53 protein.

Moreover, DCIS mixed with synchronous IBC harboured more dense stromal TILs, PD1, PDL1 and various subpopulation of lymphocytes within the stroma than pure DCIS, with the highest difference observed with stromal PD1+ (*Z* = 5.8, *p* < 0.0001) followed by stromal CD8+ (*Z* = 5.5, *p* < 0.0001). Only stromal CD4+ cells did not show a significant statistical difference between both groups. In the context of intratumoural immune cells, there was no difference in immune cells infiltrates between pure DCIS and DCIS mixed apart from intratumoural CD20, which was more prevalent in pure DCIS (*Z* = 2.7, *p* = 0.007) and PD1, which was more dense in DCIS associated with IBC (*Z* = 2.2, *p* = 0.025). Fig. 2b and Supplementary Fig. 2 demonstrate the differences in various immune cell markers between both groups.

### Association of immune cells subpopulations and immune checkpoint proteins with other clinicopathological parameters in pure DCIS cohort

Higher expression of the majority of markers, either stromal or within the intratumoural comportment, was associated with hormonal receptor negativity. The exceptions were intratumoural CD20+ cells, which were associated with ER and PR positivity, and intratumoural CD3+, CD8+ and CD4+, which showed no association with ER or PR. High expression of all markers within the stroma, and intratumoural expression of PD1 and FOXP3, was associated with higher nuclear grade, HER2 positivity, and higher expression of HIF-1α. DCIS with smaller size and lower expression of HIF-1α showed higher expression of intratumoural CD20+ lymphocytes. It was also observed that dense infiltration of PD1, CD3, CD8, CD20 and FOXP3 in the stromal region was significantly associated with the presence of comedo-type necrosis. Supplementary Table 4a–d shows the correlation between various immune cells/checkpoint markers and the clinicopathological parameters in the pure DCIS cohort.

### Copy number variation and TP53 mutations

**Fig. 3:**
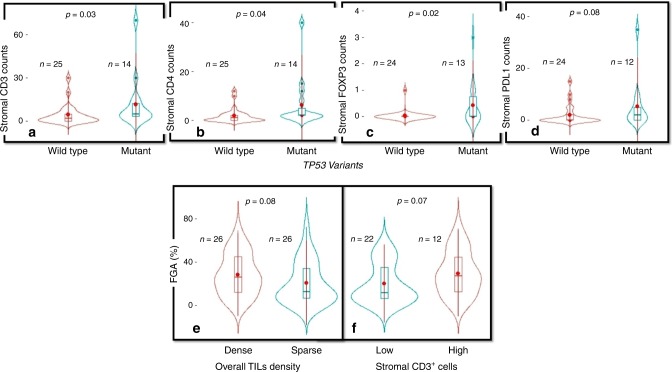
Associations between TILs and underlying genomic changes. Violin plots showing the association between *TP53* variants and **a** stromal CD3+ cells, **b** stromal CD4+ cells, **c** stromal FOXP3+ cells and **d** stromal PDL1 + TILs. The lower figures show the association between fraction of genome altered and **e** overall TILs density and **f** stromal CD3+ cells. The central red dots represent the mean, boxes represent the interquartile range, central line represent the median and whiskers shows the 95% confidence interval.

Higher FGA was associated with higher tumour grade (*p* = 0.002), presence of comedo type necrosis (*p* = 0.0003), ER negativity (*p* = 0.009), HER2 positivity (*p* = 0.0003) and presence of at least one deleterious/missense *TP53* variant (*p* < 0.0001); (Supplementary Fig. 3). Additionally, a higher NTAI was associated with high grade DCIS (*p* = 0.008), presence of comedo type necrosis (*p* = 0.006), HER2 positivity (*p* = 0.03), and with presence of deleterious/missense *TP53* variants (*p* = 0.002). The median FGA in pure DCIS with dense TIL was 26% compared to 12% in cases with sparse TILs, however the difference was not statistically significant (*p* = 0.08, Fig. 3). Similar results were observed with stromal CD3 whereas median FGA in pure DCIS with dense CD3+ cells was 26% compared to 10% in cases with sparse CD3+ cells (*p* = 0.07, Fig. 3). No association between NTAI and TILs density nor immune cell/checkpoint proteins was observed. However, tumours carrying somatic deleterious/missense *TP53* variants showed higher counts of stromal CD3+ (*p* = 0.031), CD4+ (*p* = 0.04), and FOXP3+ (*p* = 0.02); (Fig. 3).

### Outcome Analysis

**Fig. 4:**
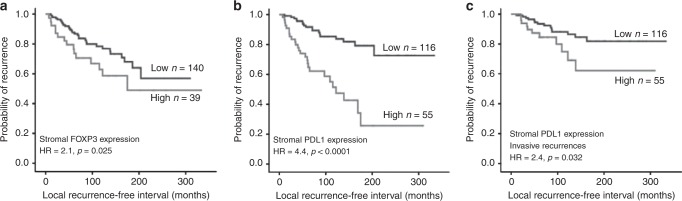
Association between TILs and DCIS outcome. Kaplan–Meier Curves showing; Higher stromal FOXP3+ cells was associated with poor outcome for all DCIS recurrences (**a**), dense stromal PDL1 expression was associated with shorter local recurrence free interval (LRFI) either for all recurrences (**b**), or invasive recurrences (**c**). All analyses were carried out on patients treated with breast conserving surgery with or without radiotherapy.

High expression of stromal FOXP3+ and stromal PDL1+ were the sole markers associated with all recurrences in DCIS patients treated with BCS (HR = 2.1, 95%CI = 1.1–3.7, *p* = 0.025 and HR = 4.4, 95%CI = 2.4–8.1, *p* < 0.0001, respectively); Fig. 4a, b. When analysis was restricted to invasive recurrences, only stromal PDL1 was associated with shorter LRFI (HR = 2.4, 95%CI = 1.1–5.3, *p* = 0.032), Fig. 4c. As previous reported^14^; overall TILs density was associated with shorter LRFI either for all recurrences (HR = 2.9, 95%CI = 1.7–5.0, *p* = 0.0001 or invasive recurrences (HR = 2.1, 95%CI = 1.1–4.3, *p* = 0.04). No significant associations with recurrence were observed for the intratumoural infiltration of any immune cell marker with recurrence (Table 1). Outcome analysis in context of various intrinsic molecular subtypes revealed that high expression of stromal FOXP3 was associated with shorter LRFI in luminal A subtype (HR = 2.9, 95%CI = 1.1–8.1, *p* = 0.032) while higher PDL1 expression in stromal TILs was associated with shorter LRFI in luminal A (HR = 7.1, 95%CI = 2.9–12.2, *p* < 0.0001) and showing a trend of poor prognosis in luminal B (HR = 3.2, 95%CI = 0.9–8.9, *p* = 0.067) and HER2 enriched (HR = 1.8, 95%CI = 0.5–7.5, *p* = 0.080) subtypes. Other markers did not show association with outcome in different molecular classes.

**Table 1:** Univariate survival analysis between overall TILs and various immune cell markers with the local recurrence free interval in DCIS patients treated with breast conserving surgery.

Marker	All recurrences				Invasive recurrences			
	Hazard ratio	95% Confidence interval		*p*-value	Hazard ratio	95% Confidence interval		p-value
		Lower	Upper			Lower	Upper	
Overall TILs	2.9	1.7	5.0	**0.0001**	2.1	1.1	4.3	**0.040**
sCD20	0.9	0.5	1.7	0.793	0.9	0.4	1.9	0.770
sCD3	1.1	0.6	1.9	0.877	0.8	0.4	1.7	0.563
sCD8	0.9	0.5	1.8	0.992	0.5	0.2	1.2	0.109
sCD4	1.5	0.9	2.8	0.150	1.2	0.5	2.5	0.708
sFOXP3	2.1	1.1	3.7	**0.025**	1.1	0.4	2.6	0.895
sPD1	1.3	0.7	2.3	0.412	0.7	0.3	1.6	0.347
sPDL-1	4.4	2.4	8.1	**<0.0001**	2.4	1.1	5.3	**0.032**
iCD20	1.2	0.7	2.2	0.520	1.1	0.5	2.3	0.937
iCD3	1.2	0.7	2.2	0.522	0.9	0.5	2.1	0.979
iCD8	1.1	0.6	1.9	0.839	0.7	0.3	1.7	0.529
iCD4	1.2	0.7	2.1	0.572	1.1	0.5	2.2	0.985
iFOXP3	1.6	0.8	3.3	0.180	0.7	0.2	2.5	0.617
iPD1	0.9	0.4	2.1	0.766	0.7	0.2	2.5	0.652

s: Stromal, i: Intratumoral.

Significant *p* values are in bold

In multivariate survival analysis, higher expression of stromal PDL1 was associated with shorter LRFI for all DCIS recurrences (but not for invasive recurrences only) independent of other parameters of DCIS aggressiveness including age at diagnosis, DCIS size, nuclear grade, presence of comedo-type necrosis and margin status (HR = 2.9, 95% CI = 1.4–6.1, *p* = 0.005). Interestingly, dense TILs were independently associated with DCIS recurrence either when included in a model comprising FOXP3+ and PDL1+ cells, or in a model where all immune cell markers associated with DCIS recurrence were incorporated with other DCIS clinicopathological parameters. A statistically significant result was obtained for all recurrences or when the analysis was confined to invasive recurrence only. Table 2A, B summarise the various multivariate models analysed.

**Table 2:** Multivariate survival analysis (Cox regression model) of variables (with and without immune cell markers) predicting outcome in terms of ipsilateral local all recurrences (A) and invasive recurrences (B) in patients treated by breast conserving surgery in pure DCIS.

Parameters	Hazard ratio (HR)	95% confidence interval (CI)		*p*-value
		Lower	Upper	
(A) All recurrences				
*Conventional clinicopathological parameters associated with high risk DCIS*				
Patient age	0.4	0.2	0.8	**0.006**
DCIS presentation^a^	1.5	0.9	2.4	0.111
DCIS size	1.5	1.1	2.1	**0.040**
DCIS nuclear Grade	1.9	1.3	2.7	**0.001**
Comedo necrosis	0.6	0.4	0.9	**0.049**
Margin status	0.8	0.7	0.9	**0.004**
*TILs density and other clinicopathological parameters*				
Patient age	0.4	0.1	0.8	**0.023**
DCIS presentation	1.5	0.9	3.8	0.395
DCIS size	0.8	0.4	1.6	0.498
DCIS nuclear Grade	1.2	0.6	2.4	0.662
Comedo necrosis	0.6	0.3	1.3	0.196
Margin status	0.9	0.9	1.1	0.637
Overall TILs density	7.0	2.8	1.2	**0.024**
Dense stromal FOXP3+ cells	1.7	0.7	0.3	0.453
Dense stromal PDL1+ cells	6.8	3.1	1.4	**0.004**
*Overall TILs density with stromal FOXP3+ and stromal PDL1+ inflammatory cells*				
Overall TILs density	3.5	1.5	8.2	**0.003**
Dense stromal FOXP3+ cells	0.8	0.4	1.7	0.554
Dense stromal PDL1+ cells	2.9	1.4	6.1	**0.005**

(B) Invasive recurrence				
Patient age	0.5	0.2	1.3	0.156
DCIS presentation	1.4	0.8	2.6	0.245
DCIS size	1.8	1.1	2.4	**0.013**
DCIS nuclear Grade	1.9	1.1	3.0	**0.013**
Comedo necrosis	0.7	0.4	1.3	0.274
Margin status	0.9	0.8	1.1	0.075
*TILs density and other clinicopathological parameters*				
Patient age	0.7	0.2	2.4	0.527
DCIS presentation	2.2	0.6	7.5	0.206
DCIS size	1.7	0.6	4.7	0.301
DCIS nuclear Grade	2.6	0.8	8.2	0.111
Comedo necrosis	0.7	0.2	2.0	0.497
Margin status	0.9	0.9	1.1	0.877
Overall TILs density	2.1	1.2	5.5	**0.029**
Dense stromal FOXP3+ cells	0.3	0.1	1.4	0.143
Dense stromal PDL1+ cells	1.7	0.6	5.1	0.307
*Overall TILs density with stromal FOXP3+ and stromal PDL1+ inflammatory cells*				
Overall TILs density	2.8	1.1	7.9	**0.045**
Dense stromal FOXP3+ cells	0.4	0.1	1.5	0.176
Dense stromal PDL1+ cells	1.7	0.6	4.8	0.318

Significant *p* values are in bold.

^a^DCIS presentation refers to the method of DCIS diagnosis either through the mammography screening program or symptomatic presentation.

Moreover, high TILs density and high stromal PDL1+ expression could categorise high risk DCIS defined as high grade DCIS and >15 mm in size into two distinct groups with different outcome; whereas high TILs and stromal PDL1 + cells were associated with shorter LRFI in these patients (HR = 3.2, 95%CI = 1.1–9.5, *p* = 0.039, and *HR* = 2.5, 95%CI = 1.1–6.3, *p* = 0.044, respectively) (Supplementary Fig. 4).

## Discussion

The importance of the tumour microenvironment dynamics during the neoplastic transformation of breast epithelial tissue is increasingly being recognised and is considered as a hallmark of cancer.^3,39,40^ However, the effect of infiltrating lymphocytes in various malignancies including DCIS is controversial^14–16,41–43^ and the role of PD1 and PDL1 proteins in DCIS remains to be defined. Through this study, we aimed to characterise TIL subtypes and their potential prognostic role in DCIS. The dominance of CD3+ cells, including CD4+ and CD8+ subsets, indicates the immunologically active state of these early tumours, which could suggest an active chemokine secretion recruitment mechanism.^44^ Apart from cell-mediated immunity, an active humoral immune response is also indicated with the high expression of CD20+ cells. This result suggests that maturation of B lymphocytes may occur at an early stage of tumour development.^45^ In comparison to pure DCIS, DCIS with synchronous IBC had notably increased levels of CD8+, CD20+, FOXP3+, PD1+ and PDL1+ cells. Since FOXP3 is the single most accurate marker of regulatory T cells (Tregs),^46^ the high expression of FOXP3+ cells in a proportion of DCIS in addition to high expression of PDL1 suggests an immunosuppressive environment being created simultaneously in early tumour development, in concordance with previous studies.^47–49^ A recent review by Chen et al. summarised findings from previous work that support our study findings, such as high grade DCIS harbouring more TILs than low grade lesions and being associated with underlying genetic alterations.^50^

We observed a significantly decreased recurrence-free survival in patients with a higher stromal infiltration of FOXP3. This suppressive environment appears to be particularly important for differentiating outcome in luminal A tumours, in which both FOXP3+ and PDL1+ were associated with poor outcome. FOXP3 was also described as an independent negative prognostic marker in IBC.^51^ Higher infiltration of FOXP3+ cells also predicts poor overall survival in ovarian carcinoma where release of the chemokine CCL22 in the tumour microenvironment by tumour associated macrophages facilitates the recruitment of FOXP3+ Tregs and hence, the suppression of cytotoxic T cells.^52^ Another mechanism by which FOXP3 aids in tumour recurrence could be by binding to the upstream region of transcription start site of chemokines such as CCR7 and CXCR4.

The crucial role of the PD1/PDL1 pathway in the attenuation of T cell-mediated immunity is well established.^53^ We observed that high expression of stromal PDL1, but not PD1, was associated with a shorter recurrence free interval. High expression of PDL1 is associated with a poorer outcome in pancreatic cancer, renal cell carcinoma and breast cancer.^54–56^ Similarly, high expression of PD1 is associated with poorer outcome in renal cell carcinoma and breast cancer.^57,58^ This association can be explained by the attenuation of the cytotoxic function of T lymphocytes in the presence of active PD1/PDL1 interactions thus aiding the tumour in its escape mechanisms.^17,59^ Another mechanism that might be at play is the upregulation of the PI3K pathway due to loss of PTEN or mutation of *PIK3CA* which ultimately leads to upregulation of PDL1.^60^ However, we saw no association of *PIK3CA* mutations with PDL1 positivity.

Despite the associations of an immunosuppressive microenvironment in DCIS with recurrence, in our multivariate analysis, overall TIL density was the strongest independent predictor for invasive recurrence. Since we did not find any significant associations of dense intratumoural infiltration of any immune cell with recurrence, we consider that assessment of stromal infiltration of different subtypes of TILs is a better method of TILs evaluation for outcome analysis in DCIS.^14^ It is similarly recommended to assess stromal TILs in IBC, but in IBC TILs are strongly associated with better outcome, particularly in TNBC.^38^ The contradictory association of TILs as a good prognostic marker in IBC and as a poor prognostic marker in DCIS could support a hypothesis that direct contact between invasive tumour cells and immune cells leads to destruction of tumour cells and hence better prognosis, while in DCIS, a dense inflammatory reaction could provide a way for destruction of the surrounding myoepithelial layer^61^ and basement membrane. This facilitation of tumour cell infiltration to the surrounding stroma^62^ would be enhanced by the immunosuppressive proteins produced by the tumour cells to escape destruction by host immunity and guarantee maintenance of the invasive tumour cells.^49^

An alternative (and not necessarily exclusive) explanation could be that a heightened immune response in DCIS and its association with poor prognosis reflects an underlying aggressive breast tumour biology. Our findings regarding the significant association of the stromal immune markers with higher grade, HER2 positivity and hormone receptor negativity has been reported previously in DCIS^63^ and IBC.^38^ The ability to recruit more inflammatory cells through production of neoantigens and chemokines, as well as their ability to modulate the immunosuppressive microenvironment to evade the host immune response, combine to develop the immune resistance shown by these tumours and lead to still further aggressive behaviour.^64^ ER negative DCIS are more proliferative and genomically unstable, hence stimulate a stronger attack from the immune cells. The association of dense infiltration in HER2+ tumours and TNBC can be best interpreted as a pro-tumorigenic function of TILs especially due to higher infiltration of Tregs.^10,63^ Our findings regarding the association of PD1 and PDL1 in TILs with higher tumour grade, were supported by the findings of Muenst et al.^58^ This association with higher grade emphasises the role of PD1/PDL1 pathway adopted by DCIS to evade anti-tumour immunity and spread aggressively. Higher expression of PDL1 in TNBC has also been reported in other studies.^60,65^ The association of more proliferative tumours such as HER2 enriched, TNBC and basal like tumours, with high expression of PDL1 could be due to the increased immunogenicity of these tumours and increased presence of neoantigens.

All solid tumours elicit an inflammatory response that is critical for the recruitment of immune cells, tumour cell proliferation, survival, and angiogenesis. Tumours initiate these responses through several mechanisms including hypoxia and derived factors.^66^ Hypoxia was reported to shape the immunosuppressive microenvironment of the tumour.^67^ The positive correlation between HIF1a and FOXP3 in our study supports such a hypothesis and shows the crosstalk between tumour microenvironment components in DCIS progression. This finding is supported other studies that showed tight interaction between hypoxia and the tumour inflammatory process.^66,68^

DCIS harbouring dense inflammatory cells was reported to have more copy number alteration^15^ and higher Oncotype DX score^69^ than those with low TILs density, which reflect the underlying biological association between TILs density and DCIS behaviour. As expected, in our study high risk DCIS (high grade, comedo type necrosis, hormonal receptor negativity and HER2 positivity) showed a higher degree of genomic instability represented by an increased burden chromosomal gains and losses and the presence of deleterious/missense *TP53* mutations.^70^ In general, genomic instability leading to high risk DCIS lesions might promote T cell activation (CD3 and CD4) and recruitment of regulatory T cells (FOXP3) as suggested by our data. These findings are supported by Hendry *et al*, who concluded that tumour immune-editing and evasion of the immune system could be a key step in progression from DCIS into IBC.^15^ Our data lacked sufficient numbers of cases analysed for mutation and CNVs to evaluate the interaction of these events with the immune context in terms of patient outcome.

Our findings could refine high risk DCIS into two distinct groups based on TILs density and stromal PDL1 expression, where high risk cases with dense TILs or higher stromal PDL1+ cells behaved more aggressively and had higher recurrence rate than high risk DCIS with lower TILs or stromal PDL1+ cells. This observation reflects the role of immune microenvironment in DCIS risk stratification and management guidance. Implementation of TILs evaluation in clinical practice could avoid further management, i.e. unnecessary postoperative radiotherapy in a subgroup of patients.

In our study, evaluation of TILs in the routine H&E sections was the only inflammatory related tool that showed independent association with DCIS recurrence, especially with invasive recurrence. Taken together, assessment of TILs in H&E sections could provide a robust prognostic data with no need for further specific immune cell markers assessment.

## Conclusion

This study highlights the importance of the immune microenvironment playing a role in DCIS behaviour. Subsets of TILs, immune checkpoint proteins and their distribution should be carefully analysed for patient risk assessment. Since patient risk stratification has been difficult due to conflicting evidence regarding TILs affecting DCIS prognosis, this study provides a more uniform inference with the higher infiltration of FOXP3+ and PDL1 immune cells being associated with poor outcome. Further mechanistic studies to clarify the crosstalk between DCIS tumour cells and the immune microenvironment are warranted.

### Limitation of the study

This study was conducted on TMA sections which may underestimate TILs infiltration although we did compare the distribution between TMA cores and full-face sections which showed comparability. In addition, due to the small number of recurrence events in patients treated with RT or when the cohort was split by molecular class or risk groups (high vs. low risk DCIS), further studies of TILs in context of these limitations are recommended. Also, the role of hormonal therapy is overlooked here as none of the study patients received such therapy. Data from genomic assessment was only available for a small subset of tumours limiting statistical power and the capability to conduct sub-group analysis (e.g. stratification by receptor status or grade), which might have weakened associations between TIL subtypes and genetic features.

## Supplementary information

Supplementary Tables and Figures

## Data Availability

The authors confirm the data that has been used in this work is available on reasonable request.

## References

[1] Ernster VL, Ballard-Barbash R, Barlow WE, Zheng Y, Weaver DL, Cutter G (2002). Detection of ductal carcinoma in situ in women undergoing screening mammography. J. Natl Cancer Inst..

[2] Groen EJ, Elshof LE, Visser LL, Th EJ, Winter-warnars HAO, Lips EH (2017). Finding the balance between over- and under-treatment of ductal carcinoma in situ (DCIS). Breast.

[3] Artacho-Cordón A, Artacho-Cordón F, Ríos-Arrabal S, Calvente I, Núñez MI (2012). Tumor microenvironment and breast cancer progression: a complex scenario. Cancer Biol. Ther..

[4] Schreiber RD, Old LJ, Smyth MJ (2011). Cancer immunoediting: integrating immunity’s roles in cancer suppression and promotion. Science.

[5] Emens LA (2012). Breast cancer immunobiology driving immunotherapy: vaccines and immune checkpoint blockade. Expert Rev. Anticancer Ther..

[6] Cruz-merino LD, Barco-sánchez A, Carrasco FH, Fernández EN, Benítez AV, Molina JB (2013). New insights into the role of the immune microenvironment in breast carcinoma. Clin. Dev. Immunol..

[7] Nelson BH (2010). CD20+ B cells: the other tumor-infiltrating lymphocytes. J. Immunol..

[8] Kotoula V, Chatzopoulos K, Lakis S, Alexopoulou Z (2016). Tumors with high-density tumor infiltrating lymphocytes constitute a favorable entity in breast cancer: a pooled analysis of four prospective adjuvant trials. Oncotarget.

[9] García-martínez E., Gil G. L., Benito A. C., González-billalabeitia E., Angeles M., Conesa V. et al. Tumor-infiltrating immune cell profiles and their change after neoadjuvant chemotherapy predict response and prognosis of breast cancer. *Breast Cancer Res*. 10.1186/s13058-014-0488-5. (2014) 10.1186/s13058-014-0488-5 PMC4303200 25432519

[10] Loi S, Michiels S, Salgado R, Sirtaine N, Jose V, Fumagalli D (2014). Tumor infiltrating lymphocytes are prognostic in triple negative breast cancer and predictive for trastuzumab benefit in early breast cancer: results from the FinHER trial. Ann. Oncol..

[11] Denkert C, von Minckwitz G, Brase JC, Sinn BV, Gade S, Kronenwett R (2015). Tumor-infiltrating lymphocytes and response to neoadjuvant chemotherapy with or without carboplatin in human epidermal growth factor receptor 2-positive and triple-negative primary breast cancers. J. Clin. Oncol..

[12] Adams S, Gray RJ, Demaria S, Goldstein L, Perez EA, Shulman LN (2014). Prognostic value of tumor-infiltrating lymphocytes in triple-negative breast cancers from two phase III randomized adjuvant breast cancer trials: ECOG 2197 and ECOG 1199. J. Clin. Oncol..

[13] Ibrahim EM, Al-Foheidi ME, Al-Mansour MM, Kazkaz GA (2014). The prognostic value of tumor-infiltrating lymphocytes in triple-negative breast cancer: a meta-analysis. Breast Cancer Res. Treat..

[14] Toss MS, Miligy I, Al-Kawaz A, Alsleem M, Khout H, Rida PC (2018). Prognostic significance of tumor-infiltrating lymphocytes in ductal carcinoma in situ of the breast. Mod. Pathol..

[15] Pruneri G, Lazzeroni M, Bagnardi V, Tiburzio GB, Rotmensz N, DeCensi A (2017). The prevalence and clinical relevance of tumor-infiltrating lymphocytes (TILs) in ductal carcinoma in situ of the breast. Ann. Oncol..

[16] Van Bockstal M, Lambein K, Gevaert O, De Wever O, Praet M, Cocquyt V (2013). Stromal architecture and periductal decorin are potential prognostic markers for ipsilateral locoregional recurrence in ductal carcinoma in situ of the breast. Histopathology.

[17] Freeman GJ, Long Aj Fau - Iwai Y, Iwai Y, Fau - Bourque K, Bourque K, Fau - Chernova T, Chernova T, Fau - Nishimura H, Nishimura H, Fau - Fitz LJ (2000). Engagement of the PD-1 immunoinhibitory receptor by a novel B7 family member leads to negative regulation of lymphocyte activation. J. Exp. Med..

[18] Keir ME, Liang SC, Guleria I, Latchman YE, Qipo A, Albacker LA (2006). Tissue expression of PD-L1 mediates peripheral T cell tolerance. J. Exp. Med.

[19] Wang X, Teng F, Kong L, Yu J (2016). PD-L1 expression in human cancers and its association with clinical outcomes. OncoTargets Ther..

[20] Topalian SL, Hodi FS, Brahmer JR, Gettinger SN, Smith DC, McDermott DF (2012). Safety, activity, and immune correlates of anti-PD-1 antibody in cancer. N. Engl. J. Med..

[21] Miligy IM, Gorringe KL, Toss MS, Al-Kawaz AA, Simpson P, Diez-Rodriguez M (2018). Thioredoxin-interacting protein is an independent risk stratifier for breast ductal carcinoma in situ. Mod. Pathol..

[22] Lester SC, Bose S, Chen YY, Connolly JL, de Baca ME, Fitzgibbons PL (2009). Protocol for the examination of specimens from patients with ductal carcinoma in situ of the breast. Arch. Pathol. Lab Med..

[23] Hammond ME, Hayes DF, Wolff AC, Mangu PB, Temin S (2010). American society of clinical oncology/college of american pathologists guideline recommendations for immunohistochemical testing of estrogen and progesterone receptors in breast cancer. J. Oncol. Pract..

[24] Rakha EA, Pinder SE, Bartlett JM, Ibrahim M, Starczynski J, Carder PJ (2015). Updated UK recommendations for HER2 assessment in breast cancer. J. Clin. Pathol..

[25] Goldhirsch A, Wood WC, Coates AS, Gelber RD, Thurlimann B, Senn HJ (2011). Strategies for subtypes-dealing with the diversity of breast cancer: highlights of the St. Gallen International Expert Consensus on the Primary Therapy of Early Breast Cancer 2011. Ann. Oncol..

[26] Bos R, van Diest PJ, de Jong JS, van der Groep P, van der Valk P, van der Wall E (2005). Hypoxia-inducible factor-1alpha is associated with angiogenesis, and expression of bFGF, PDGF-BB, and EGFR in invasive breast cancer. Histopathology.

[27] van der Groep P, van Diest PJ, Smolders YH, Ausems MG, van der Luijt RB, Menko FH (2013). HIF-1alpha overexpression in ductal carcinoma in situ of the breast in BRCA1 and BRCA2 mutation carriers. PloS ONE.

[28] Kader T, Goode DL, Wong SQ, Connaughton J, Rowley SM, Devereux L (2016). Copy number analysis by low coverage whole genome sequencing using ultra low-input DNA from formalin-fixed paraffin embedded tumor tissue. Genome Med..

[29] Van der Auwera GA, Carneiro MO, Hartl C, Poplin R, Del Angel G, Levy-Moonshine A (2013). From FastQ data to high confidence variant calls: the Genome Analysis Toolkit best practices pipeline. Curr. Protoc. Bioinforma..

[30] Rimmer A, Phan H, Mathieson I, Iqbal Z, Twigg SRF, Wilkie AOM (2014). Integrating mapping-, assembly- and haplotype-based approaches for calling variants in clinical sequencing applications. Nat. Genet..

[31] Koboldt DC, Zhang Q, Larson DE, Shen D, McLellan MD, Lin L (2012). VarScan 2: somatic mutation and copy number alteration discovery in cancer by exome sequencing. Genome Res..

[32] McLaren W, Gil L, Hunt SE, Riat HS, Ritchie GR, Thormann A (2016). The ensembl variant effect predictor. Genome Bio..

[33] Lek M, Karczewski KJ, Minikel EV, Samocha KE, Banks E, Fennell T (2016). Analysis of protein-coding genetic variation in 60,706 humans. Nature.

[34] Auton A, Brooks LD, Durbin RM, Garrison EP, Kang HM, Korbel JO (2015). A global reference for human genetic variation. Nature.

[35] Scheinin I, Sie D, Bengtsson H, van de Wiel MA, Olshen AB, van Thuijl HF (2014). DNA copy number analysis of fresh and formalin-fixed specimens by shallow whole-genome sequencing with identification and exclusion of problematic regions in the genome assembly. Genome Res..

[36] Kuilman T, Velds A, Kemper K, Ranzani M, Bombardelli L, Hoogstraat M (2015). CopywriteR: DNA copy number detection from off-target sequence data. Genome Biol..

[37] Boeva V, Popova T, Bleakley K, Chiche P, Cappo J, Schleiermacher G (2012). Control-FREEC: a tool for assessing copy number and allelic content using next-generation sequencing data. Bioinformatics.

[38] Rathore AS, Kumar S, Konwar R, Makker A, Negi MP, Goel MM (2014). CD3+, CD4+ & CD8+ tumour infiltrating lymphocytes (TILs) are predictors of favourable survival outcome in infiltrating ductal carcinoma of breast. Indian J. Med. Res..

[39] Campbell MJ, Baehner F, O’Meara T, Ojukwu E, Han B, Mukhtar R (2017). Characterizing the immune microenvironment in high-risk ductal carcinoma in situ of the breast. Breast Cancer Res. Treat..

[40] Doebar SC, de Monye C, Stoop H, Rothbarth J, Willemsen SP, van Deurzen CH (2016). Ductal carcinoma in situ diagnosed by breast needle biopsy: Predictors of invasion in the excision specimen. Breast.

[41] Kuroda H, Tamaru J-i, Sakamoto G, Ohnisi K, Itoyama S (2005). Immunophenotype of lymphocytic infiltration in medullary carcinoma of the breast. Virchows Arch..

[42] Iseki Y., Shibutani M., Maeda K., Nagahara H. A new method for evaluating tumor- infiltrating lymphocytes (TILs) in colorectal cancer using hematoxylin and eosin (H-E)—stained tumor sections. *PLoS ONE*. 10.1371/journal.pone.0192744. (2018) 10.1371/journal.pone.0192744 PMC5919485 29698402

[43] Matkowski R, Gisterek I, Halon A, Lacko A, Szewczyk K, Staszek U (2009). The prognostic role of tumor-infiltrating CD4 and CD8 T lymphocytes in breast cancer. Anticancer Res..

[44] Kristensen VN, Vaske CJ, Ursini-Siegel J, Van Loo P, Nordgard SH, Sachidanandam R (2012). Integrated molecular profiles of invasive breast tumors and ductal carcinoma in situ (DCIS) reveal differential vascular and interleukin signaling. Proc. Natl Acad. Sci. USA.

[45] Miligy I., Mohan P., Gaber A., Aleskandarany M. A., Nolan C. C., Diez-rodriguez M. et al. Prognostic significance of tumour infiltrating B lymphocytes in breast ductal carcinoma in situ. *Histopathology*. 10.1111/his.13217. (2017) 10.1111/his.13217 28326600

[46] Mahmoud S. M. A., Paish E. C., Lee A. H. S., Ellis I. O., Green A. R. An evaluation of the clinical significance of FOXP3 + infiltrating cells in human breast cancer. *Breast Cancer Res. Treat*. 10.1007/s10549-010-0987-8. (2011) 10.1007/s10549-010-0987-8 20556505

[47] Bates GJ, Fox SB, Han C, Leek RD, Garcia JF, Harris AL (2018). Quantification of regulatory T cells enables the identification of high-risk breast cancer patients and those at risk of late relapse. J. Clin. Oncol..

[48] Lal A, Chan L, Devries S, Chin K, Scott GK, Benz CC (2013). FOXP3-positive regulatory T lymphocytes and epithelial FOXP3 expression in synchronous normal, ductal carcinoma in situ, and invasive cancer of the breast. Breast Cancer Res. Treat.

[49] Gil Del Alcazar CR, Huh SJ, Ekram MB, Trinh A, Liu LL, Beca F (2017). Immune escape in breast cancer during in situ to invasive carcinoma transition. Cancer Disco..

[50] Chen XY, Yeong J, Thike AA, Bay BH, Tan PH (2019). Prognostic role of immune infiltrates in breast ductal carcinoma in situ. Breast Cancer Res. Treat..

[51] Merlo A., Casalini P., Carcangiu M. L., Malventano C., Triulzi T., Me S. FOXP3 Expression and overall survival in breast cancer. *J. Clin. Oncol.*10.1200/JCO.2008.17.9036. (2018) 10.1200/JCO.2008.17.9036 19255331

[52] Curiel TJ, Coukos G, Zou L, Alvarez X, Cheng P, Mottram P (2004). Specific recruitment of regulatory T cells in ovarian carcinoma fosters immune privilege and predicts reduced survival. Nat. Med..

[53] Dermani FK, Samadi P, Rahmani G, Kohlan AK, Najafi R (2019). PD-1/PD-L1 immune checkpoint: potential target for cancer therapy. J. Cell Physiol..

[54] Nomi T, Sho M, Akahori T, Hamada K, Kubo A, Kanehiro H (2007). Clinical Significance and therapeutic potential of the programmed death-1 ligand/programmed death-1 pathway in human pancreatic cancer. Clin. Cancer Res..

[55] Thompson RH, Gillett Md, Cheville JC, Lohse CM, Dong H, Webster WS (2004). Costimulatory B7-H1 in renal cell carcinoma patients: Indicator of tumor aggressiveness and potential therapeutic target. Proc. Natl Acad. Sci. USA.

[56] Trella E., Droeser R. A., Muenst S., Schaerli A. R., Gao F., Muraro M. G. et al. Expression of programmed death ligand 1 (PD-L1) is associated with poor prognosis in human breast cancer. *Breast Cancer Res. Treat*. 10.1007/s10549-014-2988-5. (2014) 10.1007/s10549-014-2988-5 PMC4180714 24842267

[57] Thompson RH, Dong H, Lohse CM, Leibovich BC, Blute ML, Cheville JC (2007). PD-1 is expressed by tumor-infiltrating immune cells and is associated with poor outcome for patients with renal cell carcinoma. Clin. Cancer Res..

[58] Muenst S, Soysal SD, Gao F, Obermann EC, Oertli D, EGillanders W (2013). The presence of programmed death 1 (PD-1)-positive tumor-infiltrating lymphocytes is associated with poor prognosis in human breast cancer. Breast Cancer Res. Treat..

[59] Schalper K. A., Velcheti V., Carvajal D., Wimberly H., Brown J., Pusztai L. et al. In Situ Tumor PD-L1 mRNA expression is associated with increased TILs and better outcome in breast carcinomas. *Clin. Cancer Res*. 10.1158/1078-0432.CCR-13-2702. (2014) 10.1158/1078-0432.CCR-13-2702 24647569

[60] Mittendorf EA, Philips AV, Meric-Bernstam F, Qiao N, Wu Y, Harrington S (2014). PD-L1 expression in triple-negative breast cancer. Cancer Immunol. Res.

[61] Man YG, Sang QX (2004). The significance of focal myoepithelial cell layer disruptions in human breast tumor invasion: a paradigm shift from the “protease-centered” hypothesis. Exp. Cell Res..

[62] Man YG, Stojadinovic A, Mason J, Avital I, Bilchik A, Bruecher B (2013). Tumor-infiltrating immune cells promoting tumor invasion and metastasis: existing theories. J. Cancer.

[63] Thompson E, Taube JM, Elwood H, Sharma R, Meeker A, Warzecha HN (2016). The immune microenvironment of breast ductal carcinoma in situ. Mod. Pathol..

[64] Ma X-J, Dahiya S, Richardson E, Erlander M, Sgroi DC (2009). Gene expression profiling of the tumor microenvironment during breast cancer progression. Breast Cancer Res..

[65] Sabatier R, Finetti P, Mamessier E, Adelaide J, Chaffanet M, Ali HR (2015). Prognostic and predictive value of PDL1 expression in breast cancer. Oncotarget.

[66] Triner D, Shah YM (2016). Hypoxia-inducible factors: a central link between inflammation and cancer. J. Clin. Invest..

[67] Chouaib S, Umansky V, Kieda C (2018). The role of hypoxia in shaping the recruitment of proangiogenic and immunosuppressive cells in the tumor microenvironment. Contemp. Oncol. (Pozn.).

[68] Sitkovsky M, Lukashev D (2005). Regulation of immune cells by local-tissue oxygen tension: HIF1 alpha and adenosine receptors. Nat. Rev. Immunol..

[69] Knopfelmacher A, Fox J, Lo Y, Shapiro N, Fineberg S (2015). Correlation of histopathologic features of ductal carcinoma in situ of the breast with the oncotype DX DCIS score. Mod. Pathol..

[70] Kastenhuber ER, Lowe SW (2017). Putting p53 in Context. Cell.

