# Supplementary material for: The prognostic significance of immune microenvironment in breast ductal carcinoma in situ
Source: Br J Cancer. 2020 Mar 17;122(10):1496–506. doi: 10.1038/s41416-020-0797-7 (PMC7217899; doi:10.1038/s41416-020-0797-7)
Supplement: Supplementary file 1 — Supplementary Tables and Figures [file 41416_2020_797_MOESM1_ESM.docx]

**Supplementary Table 1:** Clinicopathological characteristics of the study cohort

| **Parameters** | **Whole Pure DCIS cohort**  **(n= 780)**  **(n/%)** | **Pure DCIS cohort included in this study***  **(n=413)**  **(n/%)** | **Whole DCIS/IBC cohort****  **(n=239)**  **(n/%)** | **DCIS/IBC cohort included in this study**  **(n=154)**  **(n/%)** |
| --- | --- | --- | --- | --- |
| **Age**  ≤50 years  >50 years | 172 (22)  608 (78) | 113 (27)  300 (73) | 108 (45)  131 (55) | 73 (47)  81 (53) |
| **Presentation**  Screening  Symptomatic | 430 (55)  350 (45) | 186 (45)  227 (55) | NA | NA |
| **DCIS Size**  ≤20mm  >20mm | 392 (51)  381 (49) | 168 (41)  245 (59) | 88 (35)  155 (65) | 53 (34)  101 (66) |
| **Nuclear Grade**  Low  Moderate  High | 104 (13)  194 (25)  482 (62) | 54 (13)  107 (26)  252 (61) | 12 (5)  60 (25)  167 (70) | 8 (5)  38 (25)  108 (70) |
| **Comedo necrosis*****  Yes  No | 497 (64)  283 (36) | 273 (66)  140 (34) | 175 (73)  64 (27) | 111 (72)  43 (28) |
| **Management**  Breast conserving surgery  Mastectomy | 376 (48)  404 (52) | 181 (44)  232 (56) | NA | NA |
| **Estrogen receptor**  Negative  Positive | 148 (25)  439 (75) | 102 (21)  260 (79) | NA | NA |
| **Progesterone receptor**  Negative  Positive | 248 (42)  336 (58) | 166 (45)  200 (55) | NA | NA |
| **HER2 status**  Negative  Positive | 516 (80)  130 (20) | 288 (75)  96 (25) | NA | NA |
| **Molecular classes**  Luminal A  Luminal B  HER2 Enriched  Triple Negative | 248 (52)  88 (19)  64 (14)  73 (15) | 159 (49)  66 (20)  53 (16)  47 (15) | NA | NA |

***Refers to the number of cases included in the TMA used in this study as the number differs from the original cohort. The actual number of cases with informative TMA cores included in each study varies due to loss of some cores during sectioning and tissue processing prior to staining and was reduced with more markers stained.**

****DCIS component in the mixed cases**

*****Comedo necrosis was defined as presence of central acellular necrosis with nuclear debris that can be seen using low power examination. Tiny / punctate necrosis or focal apoptosis was not considered as comedo necrosis.**

**NA: No data available for the DCIS component in these cases, as the molecular classification and treatment were related to the invasive component not the DCIS component**

**Supplementary Table 2:** Different antibodies used and their staining protocols

| **Antibody** | **Clone** | **Source** | **Species** | **Antigen retrieval** | **Dilution** | **Incubation Time** |
| --- | --- | --- | --- | --- | --- | --- |
| CD3 | SP7 | Abcam | Rabbit monoclonal | Microwave in citrate PH6 | 1:300 | 24h |
| CD8 | C8/144B | Dako | Mouse monoclonal | Microwave in citrate PH6 | 1:300 | 60min |
| FOXP3 | 236A/E7 | Abcam | Mouse monoclonal | Microwave in citrate PH6 | 1:300 | 24h |
| CD20 | L26 | Dako | Mouse monoclonal | Microwave in citrate PH6 | 1:300 | 30min |
| CD4 | EPR6855 | Abcam | Rabbit monoclonal | Microwave in citrate PH6 | 1:500 | 24h |
| PD1 | EH33 | Cell signalling | Mouse monoclonal | Microwave in citrate PH6 | 1:75 | 24h |
| PDL1 | E1L3N | Cell signalling | Rabbit monoclonal | EDTA PH9, water bath heating at 100c | 1:25 | 24h |

**Supplementary Table 3:** Distribution of various TILs in the pure DCIS cohort

|  | sCD20 | iCD20 | sCD3 | iCD3 | sCD8 | iCD8 | sCD4 | iCD4 | sFOXP3 | iFOXP3 | sPD1 | iPD1 | sPDL1 | tPDL1 |
| --- | --- | --- | --- | --- | --- | --- | --- | --- | --- | --- | --- | --- | --- | --- |
| Median | 0 | 0 | 3 | 1 | 0 | 0 | 0 | 0 | 0 | 0 | 0 | 0 | 0 | 0 |
| Minimum | 0 | 0 | 0 | 0 | 0 | 0 | 0 | 0 | 0 | 0 | 0 | 0 | 0 | 0 |
| Maximum | 60 | 40 | 100 | 50 | 30 | 20 | 60 | 25 | 25 | 10 | 40 | 15 | 35 | 100 |
| **% Pure DCIS with high expression** | **35** | **26** | **46** | **50** | **41** | **37** | **44** | **41** | **20** | **16** | **39** | **14** | **31** | **2** |

Numbers represent average number of cells / duct except PDL1 in the tumour which represent the percentage of positive cells. s; stromal, i; intratumoural and t; tumour epithelial cells

**Supplementary Table 4 (a-d):** Correlation between immune cell markers, immune checkpoints with the clinicopathological parameters in pure DCIS cohort

| a)  Parameters | Stromal CD20 +ve cells | | χ^2^  (*p*-value) | Intratumour CD20 +ve cells | | χ^2^  (*p*-value) | Stromal CD3 +ve cells | | χ^2^  (*p*-value) | Intratumour CD3 +ve cells | | χ^2^  (*p*-value) |
| --- | --- | --- | --- | --- | --- | --- | --- | --- | --- | --- | --- | --- |
|  | Low  (N=268)  N. (%) | High  (n=145)  N. (%) |  | Low  (N=305)  N. (%) | High  (n=108)  N. (%) |  | Low  (N=207)  N. (%) | High  (n=203)  N. (%) |  | Low  (N=207)  N. (%) | High  (n=203)  N. (%) |  |
| **Age**  ≤50 years  >50 years | 66 (25)  202 (75) | 47 (32)  98 (68) | 2.9  (0.090) | 79 (26)  226 (74) | 34 (31)  74 (69) | 1.2  (0.264) | 55 (25)  165 (75) | 56 (30)  134 (70) | 1.1  (0.309) | 52 (25)  155 (75) | 59 (29)  144 (71) | 0.8  (0.369) |
| **Presentation**  Screening  Symptomatic | 126 (47)  142 (53) | 60 (41)  85 (59) | 1.2  (0.272) | 138 (45)  167 (55) | 48 (44)  60 (56) | 0.021  (0.886) | 101 (50)  119 (50) | 84 (44)  106 (56) | 0.1  (0.730) | 92 (44)  115 (56) | 93 (46)  110 (54) | 0.1  (0.781) |
| **DCIS Size**  ≤20mm  >20mm | 109 (41)  158 (59) | 59 (41)  85 (59) | 0.1  (0.977) | 116 (38)  187 (62) | 52 (48)  56 (52) | 3.2  (0.073) | 92 (42)  128 (58) | 75 (40)  113 (60) | 0.2  (0.694) | 74 (36)  132 (64) | 93 (46)  109 (54) | 4.3  **(0.038)** |
| **Nuclear Grade**  Low  Moderate  High | 43 (15)  81 (28)  168 (57) | 9 (6)  27 (19)  85 (75) | 19.1  **(<0.0001)** | 36 (12)  81 (27)  188 (61) | 16 (15)  27 (25)  65 (60) | 0.676  (0.713) | 41 (18)  71 (33)  108 (49) | 10 (5)  35 (18)  145 (77) | 34.5  **(<0.0001)** | 26 (13)  57 (28)  124 (59) | 25 (12)  49 (24)  129 (64) | 0.7  (0.711) |
| **Comedo necrosis**  Yes  No | 163 (61)  105 (39) | 110 (76)  35 (24) | 9.5  **(0.002)** | 207 (68)  98 (32) | 66 (61)  42 (39) | 1.6  (0.202) | 132 (60)  88 (40) | 139 (73)  51 (27) | 7.9  **(0.005)** | 140 (68)  67 (32) | 131 (65)  72 (35) | 0.4  (0.507) |
| **Estrogen receptor**  Negative  Positive | 45 (19)  188 (81) | 57 (44)  72 (56) | 25.4  **(<0.0001)** | 83 (32)  180 (68) | 19 (19)  80 (81) | 5.4  **(0.020)** | 28 (15)  160 (85) | 73 (42)  99 (58) | 33.8  **(<0.0001)** | 53 (30)  124 (70) | 48 (26)  135 (74) | 0.6  (0.433) |
| **Progesterone receptor**  Negative  Positive | 87 (37)  151 (63) | 79 (62)  49 (38) | 21.3  **(<0.0001)** | 132 (49)  135 (51) | 34 (34)  65 (66) | 6.6  **(0.010)** | 62 (32)  131 (68) | 103 (61)  67 (39) | 29.5  **(<0.0001)** | 86 (48)  94 (52) | 79 (43)  104 (57) | 0.8  (0.378) |
| **HER2 status ***  Negative  Positive | 202 (80)  49 (20) | 86 (65)  47 (35) | 11.6  **(0.001)** | 205 (73)  76 (27) | 83 (81)  20 (19) | 2.3  (0.126) | 170 (83)  34 (17) | 115 (65)  62 (35) | 16.9  **(<0.0001)** | 144 (75)  49 (25) | 141 (75)  47 (25) | 0.1  (0.930) |
| **Molecular classes**  Luminal A  Luminal B  HER2 Enriched  Triple Negative | 119 (58)  42 (20)  22 (11)  23 (11) | 40 (34)  24 (20)  31 (26)  24 (20) | 24.1  **(<0.0001)** | 106 (46)  45 (19)  43 (19)  38 (16) | 53 (57)  21 (23)  10 (12)  9 (10) | 6.6  (0.086) | 101 (62)  32 (20)  14 (9)  14 (9) | 57 (35)  34 (21)  39 (24)  32 (20) | 31.1  **(<0.0001)** | 71 (46)  31 (20)  27 (18)  25 (16) | 87 (52)  35 (21)  26 (15)  21 (12) | 1.5  (0.674) |
| **HIF1a expression**  Low  High | 207 (83)  44 (17) | 90 (63)  53 (37) | 18.7  **(<0.0001)** | 207 (72)  80 (28) | 90 (84)  17 (16) | 6.3  **(0.014)** | 171 (84)  33 (16) | 126 (66)  64 (34) | 16.8  **(<0.0001)** | 144 (75)  49 (25) | 151 (76)  48 (24) | 0.1  (0.771) |

| b)  Parameters | Stromal CD4 +ve cells | | χ^2^  (*p*-value) | Intratumour CD4 +ve cells | | χ^2^  (*p*-value) | Stromal CD8 +ve cells | | χ^2^  (*p*-value) | Intratumour CD8 +ve cells | | χ^2^  (*p*-value) |
| --- | --- | --- | --- | --- | --- | --- | --- | --- | --- | --- | --- | --- |
|  | Low  (N=225)  N. (%) | High  (N=178)  N. (%) |  | Low  (N=233)  N. (%) | High  (N=170)  N. (%) |  | Low  (N=238)  N. (%) | High  (n=164)  N. (%) |  | Low  (N=253)  N. (%) | High  (n=149)  N. (%) |  |
| **Age**  ≤50 years  >50 years | 55 (24)  170 (76) | 57 (32)  121 (68) | 2.8  (0.092) | 65 (28)  168 (72) | 47 (28)  123 (72) | 0.1  (0.956) | 54 (23)  184 (77) | 57 (35)  107 (65) | 6.6  **(0.010)** | 65 (26)  188 (74) | 46 (31)  103 (69) | 1.3  (0.262) |
| **Presentation**  Screening  Symptomatic | 106 (47)  119 (53) | 75 (42)  103 (58) | 0.9  (0.363) | 103 (44)  130 (56) | 78 (46)  92 (54) | 0.1  (0.738) | 114 (48)  124 (52) | 66 (40)  98 (60) | 2.3  (0.129) | 107 (42)  146 (58) | 73 (49)  76 (51) | 1.7  (0.192) |
| **DCIS Size**  ≤20mm  >20mm | 88 (39)  137 (61) | 75 (43)  101 (57) | 0.6  (0.424) | 83 (36)  150 (64) | 80 (48)  88 (52) | 5.8  **(0.016)** | 94 (40)  144 (60) | 67 (41)  95 (59) | 0.1  (0.773) | 92 (36)  161 (64) | 69 (47)  78 (53) | 4.3  (0.038) |
| **Nuclear Grade**  Low  Moderate  High | 40 (18)  75 (34)  110 (48) | 11 (6)  29 (16)  138 (78) | 36.9  **(<0.0001)** | 29 (12)  63 (27)  141 (61) | 22 (13)  41 (24)  107 (63) | 0.4  (0.803) | 43 (19)  71 (29)  123 (52) | 7 (4)  35 (21)  123 (75) | 26.0  **(<0.0001)** | 37 (15)  72 (29)  144 (56) | 13 (9)  34 (23)  102 (68) | 5.8  (0.055) |
| **Comedo necrosis**  Yes  No | 143 (63)  82 (37) | 125 (70)  53 (30) | 2.2  (0.134) | 165 (71)  68 (29) | 103 (61)  67 (39) | 4.6  **(0.032)** | 148 (63)  90 (37) | 119 (73)  45 (27) | 4.3  **(0.037)** | 168 (66)  85 (34) | 99 (66)  50 (34) | 0.1  (0.993) |
| **Estrogen receptor**  Negative  Positive | 28 (14)  168 (86) | 73 (46)  86 (54) | 44.8  **(<0.0001)** | 61 (31)  138 (69) | 40 (26)  116 (74) | 1.1  (0.299) | 38 (19)  173 (81) | 62 (43)  82 (57) | 25.1  **(<0.0001)** | 54 (25)  165 (75) | 46 (34)  90 (66) | 3.5  (0.062) |
| **Progesterone receptor**  Negative  Positive | 66 (33)  132 (67) | 96 (60)  64 (40) | 25.0  **(<0.0001)** | 101 (49)  106 (51) | 61 (40)  90 (60) | 2.5  (0.115) | 77 (36)  139 (64) | 85 (59)  59 (41) | 19.0  **(<0.0001)** | 93 (41)  132 (59) | 69 (51)  66 (49) | 3.3  (0.071) |
| **HER2 status ***  Negative  Positive | 175 (83)  36 (17) | 107 (65)  58 (35) | 14.3  **(<0.0001)** | 159 (73)  60 (27) | 123 (78)  34 (22) | 1.6  (0.205) | 184 (82)  40 (18) | 97 (64)  54 (36) | 15.4  **(0.001)** | 182 (77)  54 (23) | 99 (71)  40 (29) | 1.6  (0.203) |
| **Molecular classes**  Luminal A  Luminal B  HER2 Enriched  Triple Negative | 110 (65)  33 (19)  13 (8)  15 (8) | 47 (31)  32 (21)  40 (27)  31 (21) | 44.4  **(<0.0001)** | 80 (45)  38 (22)  32 (18)  27 (15) | 77 (53)  27 (19)  21 (15)  19 (13) | 2.2  (0.527) | 120 (63)  31 (16)  18 (10)  21 (11) | 37 (28)  34 (26)  35 (27)  24 (19) | 40.3  **(<0.0001)** | 102 (54)  35 (18)  29 (15)  24 (13) | 55 (42)  30 (23)  24 (19)  21 (16) | 4.1  (0.260) |
| **HIF1a expression**  Low  High | 180 (85)  33 (15) | 112 (64)  63 (36) | 24.1  **(<0.0001)** | 168 (76)  53 (24) | 124 (74)  43 (26) | 0.2  (0.690) | 196 (85)  32 (15) | 97 (61)  62 (39) | 29.1  **(<0.0001)** | 188 (78)  52 (22) | 105 (71)  42 (29) | 2.4  (0.124) |

| c)  Parameters | Stromal FOXP3 +ve cells | | χ^2^  (*p*-value) | Intratumour FOXP3 +ve cells | | χ^2^  (*p*-value) |
| --- | --- | --- | --- | --- | --- | --- |
|  | Low  (N=327)  N. (%) | High  (N=79)  N. (%) |  | Low  (N=330)  N. (%) | High  (N=66)  N. (%) |  |
| **Age**  ≤50 years  >50 years | 91 (28)  236 (72) | 20 (25)  59 (75) | 0.2  (0.681) | 91 (27)  239 (73) | 20 (30)  46 (70) | 0.3  (0.593) |
| **Presentation**  Screening  Symptomatic | 143 (44)  180 (56) | 39 (49)  40 (51) | 0.7  (0.405) | 156 (46)  180 (54) | 26 (39)  40 (61) | 1.1  (0.294) |
| **DCIS Size**  ≤20mm  >20mm | 132 (41)  191 (59) | 31 (40)  46 (60) | 0.1  (0.931) | 134 (40)  201 (60) | 29 (45)  36 (55) | 0.5  (0.488) |
| **Nuclear Grade**  Low  Moderate  High | 48 (15)  90 (28)  185 (57) | 1 (1)  13 (17)  65 (82) | 19.9  **(<0.0001)** | 47 (14)  92 (27)  197 (59) | 2 (3)  11 (16)  53 (81) | 12.1  **(0.002)** |
| **Comedo necrosis**  Yes  No | 204 (63)  119 (37) | 64 (19)  15 (81) | 9.4  **(0.002)** | 218 (65)  118 (35) | 50 (76)  16 (24) | 2.9  (0.087) |
| **Estrogen receptor**  Negative  Positive | 67 (24)  214 (76) | 34 (53)  39 (47) | 15.1  **(<0.0001)** | 78 (27)  216 (73) | 23 (38)  37 (62) | 3.4  **(0.049)** |
| **Progesterone receptor**  Negative  Positive | 114 (40)  173 (60) | 49 (69)  22 (31) | 19.8  **(<0.0001)** | 127 (42)  172 (53) | 36 (61)  23 (39) | 6.8  **(0.009)** |
| **HER2 status ***  Negative  Positive | 240 (80)  64 (20) | 40 (58)  31 (42) | 13.7  **(<0.0001)** | 244 (78)  70 (22) | 36 (59)  25 (41) | 9.4  **(0.002)** |
| **Molecular classes**  Luminal A  Luminal B  HER2 Enriched  Triple Negative | 140 (55)  48 (20)  31 (12)  35 (13) | 17 (25)  17 (25)  22 (33)  11 (17) | 24.6  **(<0.0001)** | 139 (53)  46 (17)  39 (15)  37 (15) | 17 (29)  18 (31)  14 (24)  9 (16) | 12.3  **(0.006)** |
| **HIF1a expression**  Low  High | 251 (81)  58 (19) | 39 (50)  39 (50) | 32.7  **(<0.0001)** | 256 (80)  66 (20) | 34 (52)  31 (48) | 21.3  **(<0.0001)** |

| d)  Parameters | Stromal PD1 +ve cells | | χ^2^  (*p*-value) | Intratumour PD1 +ve cells | | χ^2^  (*p*-value) | Stromal PDL1 +ve cells | | χ^2^  (*p*-value) |
| --- | --- | --- | --- | --- | --- | --- | --- | --- | --- |
|  | Low  (N=241)  N. (%) | High  (n=152)  N. (%) |  | Low  (N=338)  N. (%) | High  (n=55)  N. (%) |  | Low  (N=264)  N. (%) | High  (n=119)  N. (%) |  |
| **Age**  ≤50 years  >50 years | 63 (26)  178 (74) | 47 (31)  105 (69) | 0.9  (0.328) | 95 (28)  243 (72) | 15 (27)  40 (73) | 0.1  (0.898) | 65 (25)  199 (75) | 40 (34)  79 (66) | 3.3  (0.068) |
| **Presentation**  Screening  Symptomatic | 114 (47)  127 (53) | 64 (42)  88 (58) | 0.8  (0.357) | 154 (46)  184 (54) | 24 (44)  31 (56) | 0.1  (0.790) | 128 (49)  136 (51) | 45 (38)  74 (62) | 3.8  (0.052) |
| **DCIS Size**  ≤20mm  >20mm | 97 (41)  144 (59) | 59 (39)  91 (61) | 0.6  (0.806) | 132 (39)  206 (61) | 24 (45)  29 (55) | 0.742  (0.389) | 114 (43)  150 (57) | 39 (33)  79 (67) | 3.8  (0.062) |
| **Nuclear Grade**  Low  Moderate  High | 40 (17)  83 (35)  118 (48) | 9 (6)  22 (15)  121 (79) | 37.7  **(<0.0001)** | 48 (14)  95 (28)  195 (58) | 1 (2)  10 (18)  44 (80) | 11.4  **(0.003)** | 42 (16)  79 (30)  143 (54) | 7 (6)  22 (19)  90 (75) | 16.7  **(<0.0001)** |
| **Comedo necrosis**  Yes  No | 146 (60)  95 (40) | 117 (77)  35 (23) | 11.7  **(0.001)** | 223 (66)  115 (34) | 40 (73)  15 (27) | 0.9  (0.324) | 169 (64)  95 (36) | 86 (72)  33 (28) | 2.5  (0.113) |
| **Estrogen receptor**  Negative  Positive | 32 (15)  176 (85) | 66 (46)  76 (54) | 41.6  **(<0.0001)** | 76 (25)  222 (75) | 22 (42)  30 (58) | 6.2  **(0.013)** | 46 (20)  187 (80) | 53 (49)  55 (51) | 30.8  **(<0.0001)** |
| **Progesterone receptor**  Negative  Positive | 66 (30)  149 (70) | 95 (67)  46 (33) | 47.8  **(<0.0001)** | 125 (41)  180 (59) | 36 (71)  15 (29) | 15.5  **(<0.0001)** | 82 (35)  152 (65) | 74 (68)  35 (32) | 32.4  **(<0.0001)** |
| **HER2 status ***  Negative  Positive | 187 (84)  37 (16) | 85 (60)  57 (40) | 12.9  **(<0.0001)** | 243 (77)  71 (23) | 29 (56)  23 (44) | 10.9  **(0.001)** | 199 (82)  44 (18) | 64 (58)  47 (42) | 23.4  **(<0.0001)** |
| **Molecular classes**  Luminal A  Luminal B  HER2 Enriched  Triple Negative | 117 (64)  34 (19)  14 (7)  18 (10) | 39 (29)  30 (23)  40 (30)  24 (18) | 47.4  **(<0.0001)** | 137 (51)  55 (21)  39 (15)  35 (13) | 19 (38)  9 (18)  15 (30)  7 (14) | 7.5  (0.057) | 123 (60)  37 (18)  23 (11)  21 (11) | 25 (25)  25 (25)  30 (29)  22 (21) | 38.4  **(<0.0001)** |
| **HIF1a expression**  Low  High | 191 (85)  34 (15) | 91 (61)  59 (39) | 28.1  **(<0.0001)** | 251 (78)  70 (22) | 31 (57)  23 (43) | 10.7  **(0.001)** | 208 (85)  37 (15) | 65 (55)  53 (45) | 37.9  **(<0.0001)** |

**Significant *p* values are in bold.**

DCIS; ductal carcinoma in situ, HER2; Human epidermal growth factor receptor 2, HIF1A; hypoxia inducible factor 1 alpha.

**Supplementary Figures**


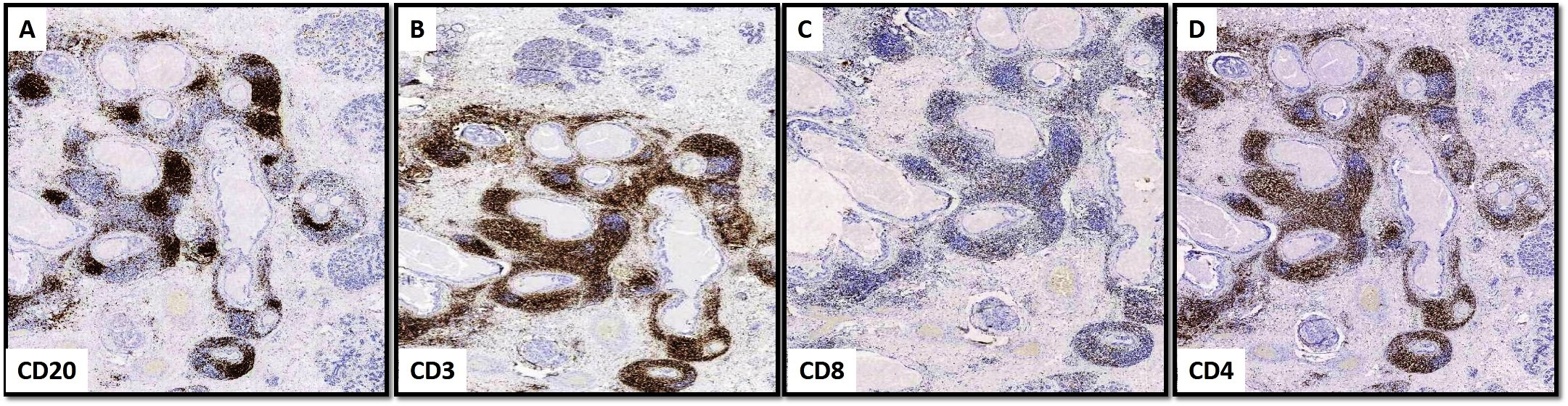


**Supplementary Figure 1**: Low power view (4x), showing distribution of CD20, CD3, CD4 and CD8 in a DCIS case. It is noticed that CD20 and CD3 are the most predominant subtypes. Also, CD4+ cells are more predominant T lymphocytes’ subtype than CD8+ cells.

| 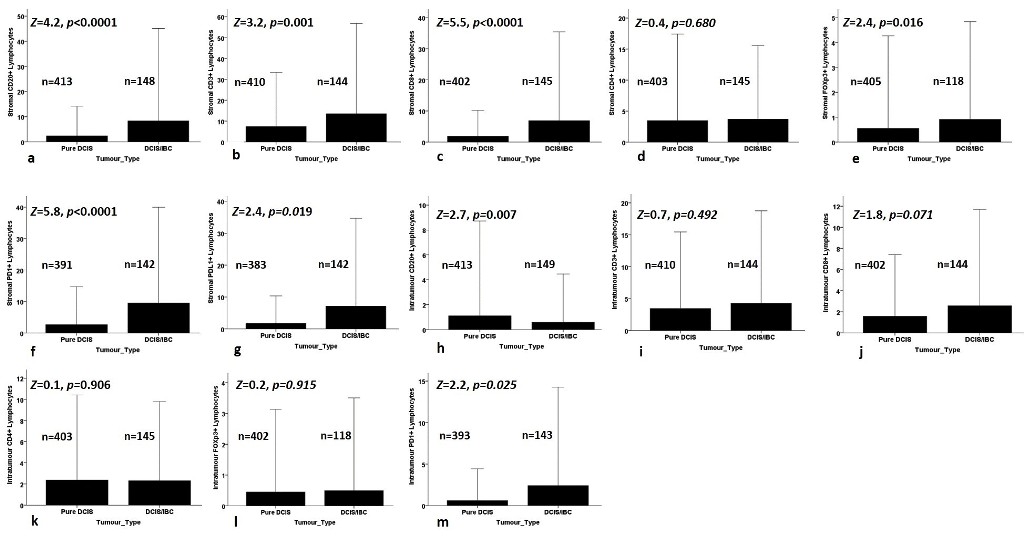 |
| --- |

**Supplementary Figure 2:** Bar charts showing the difference between expression of various immune cells and checkpoint proteins in pure DCIS and DCIS coexisting with invasive disease; a) stromal CD20+, b) stromal CD3+, c) stromal CD8+, d) stromal CD4+, e) stromal FOXP3+, f) stromal PD1+, g) stromal PDL1+, h) intratumoural CD20+, i) intratumoural CD3+, j) intratumoural CD8+, k) intratumoural CD4+, l) intratumoural FOXP3+ and m) intratumoural PD1+ lymphocytes. Error bars represent +2 standard deviation.

| 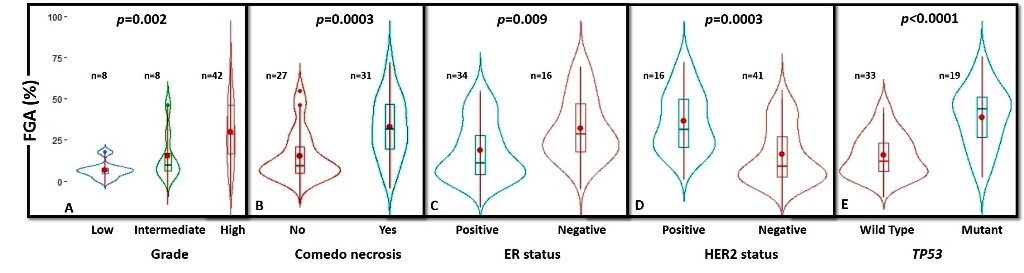 |
| --- |
| 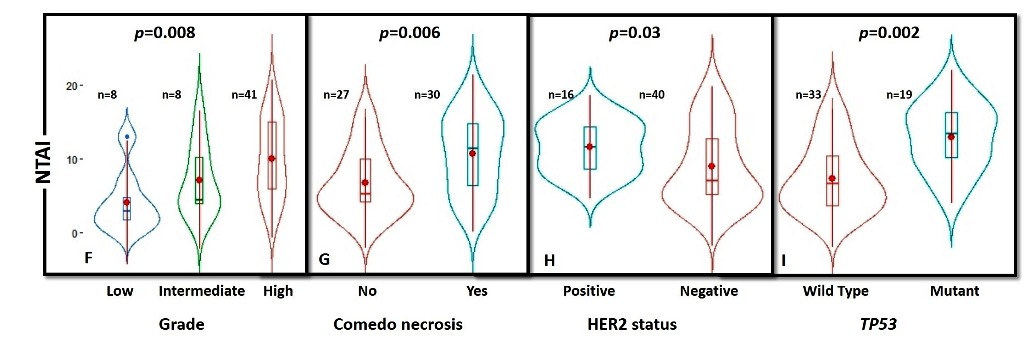 |

**Supplementary Figure 3:** Violin plots showing the association between various clinicopathological parameters, TP53 mutation with (A-E) the fraction of genomic altered (FGA) and with (F-I) number of telomeric allelic imbalances (NTAI). The central red dots represent the mean, boxes represent the interquartile range, central line represent the median and whiskers shows the 95% confidence interval.

| 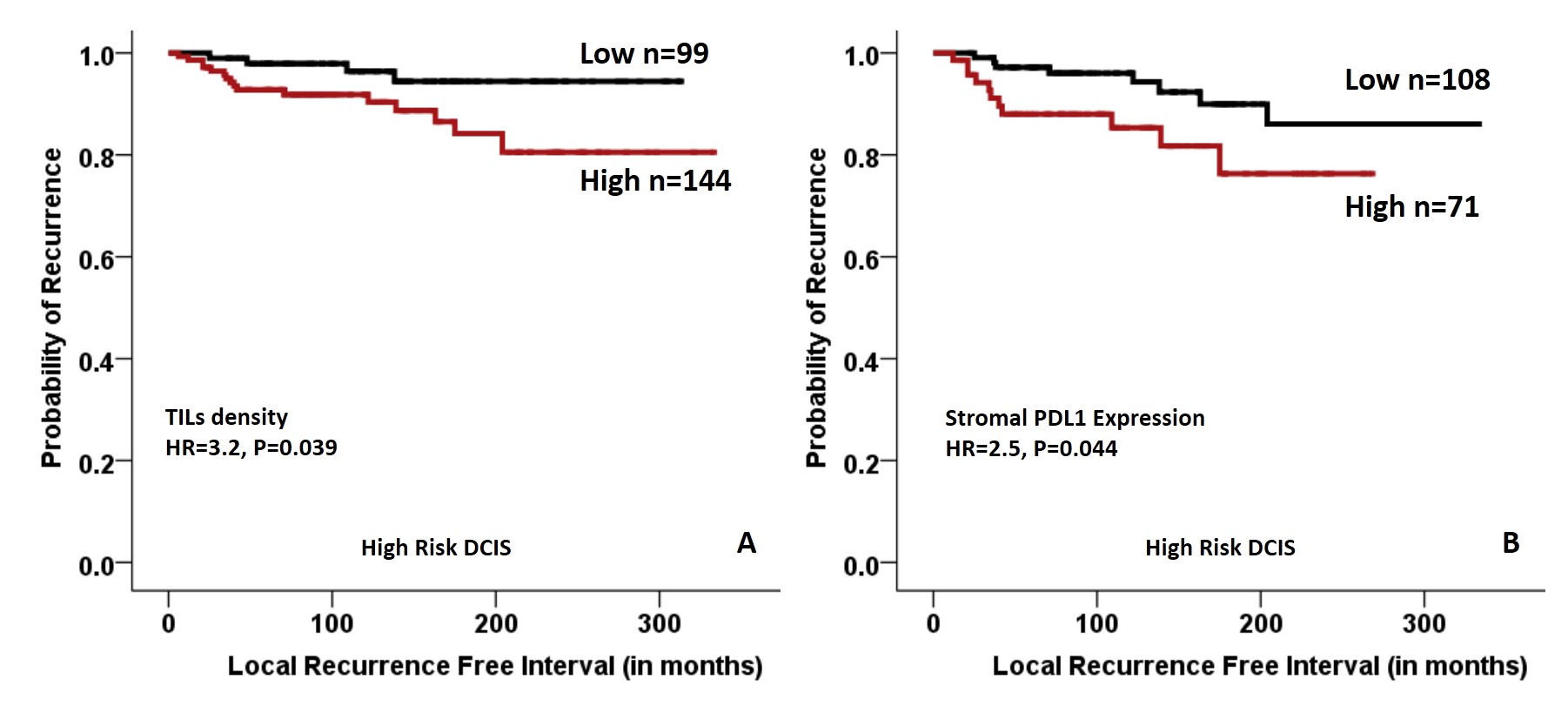 |
| --- |

**Supplementary Figure 4:** Kaplan Meier Curves showing; A) Higher TILs density is associated with shorter local recurrence free interval in high risk DCIS defined as high grade DCIS with size>1.5cm. B) High stromal PDL1 expression is associated with shorter local recurrence free interval (LRFI) in high risk DCIS.
